# Biomarker repurposing: opportunities and challenges in the evolving field of laboratory medicine

**DOI:** 10.1515/almed-2024-0158

**Published:** 2024-12-31

**Authors:** Damien Gruson, Nassiba Menghoun, Anne-Catherine Pouleur, Antoni Bayes Genis

**Affiliations:** Department of Laboratory Medicine, Cliniques Universitaires St-Lux, Université Catholique de Louvain, Brussels, Belgium; Pôle de recherche en Endocrinologie, Diabète et Nutrition, Institut de Recherche Expérimentale et Clinique, Cliniques Universitaires Saint-Luc and Université Catholique de Louvain, Brussels, Belgium; Cardiovascular Department, 70492 Cliniques universitaires Saint-Luc, Brussels, Belgium; Pôle de Recherche Cardiovasculaire (CARD), Institut de Recherche Expérimentale et Clinique (IREC), Université Catholique de Louvain (UCLouvain), Brussels, Belgium; Germans Trias Heart Institute (iCor), Pujol, Universitat Autònoma de Barcelona, CIBERCV, Barcelona, Spain

**Keywords:** CA125, biomarker, repurposing, heart failure, congestion and inflammation, clinical diagnostics

To the Editor,

The concept of drug repurposing – using existing drugs for new therapeutic purposes – has gained significant attention in recent years, offering the potential for faster, more cost-effective treatments [1], 2]. A parallel trend, biomarker repurposing, could emerge as a new field of interest in clinical research, offering innovative ways to utilize established biomarkers beyond their original applications [3]. A notable example of this is the biomarker carbohydrate antigen 125 (CA125), traditionally associated with ovarian cancer, now showing potential in the management of heart failure (HF), a disease where the value of biomarkers is well established [4], 5]. More specifically, natriuretic peptides, B-type natriuretic peptide (BNP) and N-terminal proBNP (Nt-proBNP), are part of the standard of care for the diagnosis of HF [6]. This evolution raises important questions about how biomarkers could be repurposed to provide insights into new diseases, accelerating diagnostic and therapeutic advancements.

CA125: from ovarian cancer to heart failure: Carbohydrate Antigen 125 (CA125) has long been recognized as a biomarker for the diagnosis and management of ovarian cancer, assisting in monitoring disease progression and response to treatment [7]. However, recent advances highlight its utility in other clinical contexts, particularly heart failure (HF). This glycoprotein, encoded by the MUC16 gene and synthesized by mesothelial cells, is involved in processes such as cell-mediated immune responses, inflammation, and fluid transport [4], 8]. Interestingly, these mechanisms overlap significantly between cancer and HF, suggesting shared pathways such as inflammation, congestion, and fibrosis.

In HF, CA125 levels are elevated, particularly in patients with right-sided HF or significant congestion, reflecting hemodynamic changes such as fluid retention and edema. These changes may represent a common pathophysiological mechanism shared between cancer and HF, particularly conditions characterized by mesothelial cell activation and fluid dysregulation [9]. This insight broadens the understanding of CA125 beyond its traditional role, offering a novel perspective on its involvement in systemic disease processes.

Moreover, in HF patients, CA125 has emerged as a valuable marker for monitoring disease severity and guiding treatment decisions. For example, elevated CA125 levels have been strongly correlated with congestion, inflammation, and fibrosis, as evidenced by associations with markers such as N-terminal pro-brain natriuretic peptide (NT-proBNP) and fibroblast growth factor 23 (FGF-23), particularly in heart failure with preserved ejection fraction (HFpEF) [8], 10]​. These findings underscore its potential as a prognostic tool for HF management.

Beyond ovarian cancer, the clinical utility of CA125 may extend to other neoplasms, including malignant pleural mesothelioma and other epithelial-derived tumors. Mesothelial cell involvement and fluid retention in pleural effusions suggest a potential role for CA125 as a diagnostic or prognostic marker in these conditions. Its relevance in various diseases highlights the potential for repurposing biomarkers like CA125 across diverse clinical settings, enabling clinicians to manage complex diseases such as HF and potentially other neoplasms with improved precision. This growing evidence reaffirms CA125’s role as a dynamic biomarker at the intersection of oncology and cardiology, offering a unique lens to explore the shared mechanisms between cancer and cardiovascular diseases [11].

The case for biomarker repurposing: Biomarker repurposing involves finding and validating biomarkers that were initially identified for one disease or condition and applying them to another. This approach offers multiple advantages, including the reduction of time and costs associated with developing and validating new biomarkers from scratch. Like drug repurposing, biomarker repurposing can leverage existing knowledge and infrastructure, accelerating the process of translating scientific discovery into clinical practice [2]​. In the case of CA125, its repurposing from oncology to cardiology reflects a broader trend in the medical community’s growing understanding of shared pathophysiological mechanisms across diseases. For instance, inflammation, tissue remodeling, and fibrosis are common pathways in both cancer and heart failure, providing a rationale for investigating CA125 in cardiovascular conditions [4], 10]​. By recognizing these shared mechanisms, researchers can apply biomarkers across different diseases, unlocking new diagnostic and therapeutic opportunities.

Challenges in biomarker repurposing: While biomarker repurposing presents a promising avenue, it is not without challenges. One key issue is the need for comprehensive validation in different disease contexts. For example, while CA125 has proven useful in heart failure, its role in this new context requires further validation to ensure that it provides clinically meaningful information in routine practice​ [4], 8].

Another challenge lies in understanding the biomarker’s mechanistic underpinnings. Biomarkers like CA125 may behave differently across diseases due to varying underlying pathophysiological mechanisms. In ovarian cancer, CA125 levels correlate with tumor burden, but in heart failure, they are more reflective of congestion and systemic inflammation​ [10]. Such differences necessitate rigorous mechanistic studies to ensure that repurposed biomarkers remain reliable and accurate. Furthermore, clinical implementation requires careful consideration of how a biomarker will be integrated into existing diagnostic workflows. In the case of CA125, widespread availability and cost-effectiveness make it an attractive candidate for routine use in HF, yet practical questions regarding its place in treatment algorithms and patient management protocols still need to be addressed​ [4]. While most CA125 assays bear the CE mark, compliance with IVDR regulations may necessitate reevaluation for new applications, particularly in cardiovascular diseases.

The role of artificial intelligence in biomarker and drug repurposing: Artificial intelligence (AI) and machine learning technologies are playing an increasingly important role in the field of drug repurposing, and the same principles can be applied to biomarker repurposing. AI algorithms can sift through vast amounts of biomedical data to identify potential biomarkers that may be repurposed across different diseases. This approach offers a significant advantage in rare diseases, where data are often limited​ [2]. For example, AI-driven platforms have successfully identified new applications for drugs in the treatment of rare diseases, a process that could similarly be applied to biomarkers [2]​. By analyzing patterns in patient data, including genetic, proteomic, and clinical information, AI tools could help identify new roles for existing biomarkers like CA125, accelerating the process of discovery and clinical translation.

Clinical implications and future directions: The successful repurposing of biomarkers like CA125 could have profound implications for clinical practice. In heart failure, where accurate biomarkers are critical for prognosis and treatment guidance, CA125 offers a new tool to enhance patient care. Its ability to reflect congestion and inflammation makes it a valuable complement to other established markers such as NT-proBNP [8], 10]. By incorporating CA125 into clinical protocols, physicians may be able to better stratify patients based on their risk and adjust treatments accordingly. Moreover, the concept of biomarker repurposing could be extended to other fields of medicine. As our understanding of shared disease mechanisms grows, biomarkers originally associated with one condition may find new life in others, creating new opportunities for diagnosis, monitoring, and treatment across a wide range of diseases. The principles of biomarker repurposing extend beyond CA125, with potential examples including galectin-3 in inflammation and procalcitonin in sepsis and oncology. In conclusion, the repurposing of biomarkers like CA125 represents an exciting frontier in personalized medicine. By leveraging existing biomarkers for new purposes, the medical community can expedite the development of diagnostic and therapeutic tools, ultimately improving patient outcomes across a variety of conditions. As research progresses, the integration of AI and other advanced technologies will only accelerate this process, making biomarker repurposing a key element of future healthcare innovation.
